# Impact of diabetes mellitus on functional exercise capacity and pulmonary functions in patients with diabetes and healthy persons

**DOI:** 10.1186/s12902-018-0328-1

**Published:** 2019-01-03

**Authors:** Krzysztof Kuziemski, Wojciech Słomiński, Ewa Jassem

**Affiliations:** 1 Department of Pulmonology and Allergology, Faculty of Medicine, Medical University of Gdansk, Dębinki 7, 80-211 Gdańsk, Poland; 2 Nordea Bank Abp S.A., Satamaradankatu 5, FI-00020, Helsinki, Finland

**Keywords:** 6-min walk test, Physical capacity, Pulmonary function tests, Spirometry, Lung diffusion capacity

## Abstract

**Background:**

Chronic diabetic complications may afflict all organ tissues, including those of the respiratory system**.** The six-minute walk test (6MWT) is an alternative and widely used method of assessing functional capacity and is simple to perform. However, to our knowledge, the impact of diabetes mellitus on 6MWT performance has not been investigated previously. This research aimed to compare the functional exercise capacity and pulmonary functions in patients with diabetes and in healthy persons.

**Methods:**

The study included 131 participants: 64 patients with type 1 and 2 diabetes mellitus (DM) and 67 healthy participants (CG). All of the participants were nonsmoking and did not have pulmonary disorders that affected the pulmonary function tests or 6MWT. Metabolic parameters and biochemical markers of inflammation were assessed. Full lung function tests and a 6MWT were performed.

**Results:**

In the DM group, the walking distance was 109 m shorter than that in the CG (*P* < 0.001). Moreover, compared to the CG, the DM group showed lower values of forced expiratory volume in one second (FEV1 (l) 3.6 vs. 2.8, *P* < 0.001) and total lung capacity (TLC (l) 6.6 vs. 5.6, *P* < 0.001), as well as a decrease in diffusion capacity (DLCO (mmol/min/kPa), 10.0 vs. 8.6, *P* < 0.001).

**Conclusions:**

The 6MWT is a valuable test that complements the assessment of daily physical capacity in patients with diabetes, irrespective of type. Pulmonary function and the capacity for physical exertion varied between patients with diabetes mellitus and the healthy participants in the CG.

## Background

Diabetes is a serious, chronic disease and, as a public health problem classified as a lifestyle disease, is an important social, epidemiological and economical problem. The number of people with diabetes mellitus increased from 108 million in 1980 to 422 million in 2014 [1]. The global prevalence of diabetes among adults over 18 years of age has nearly doubled since 1980, rising from 4.7 to 8.5% in the adult population in 2014. In 2015, an estimated 1.6 million deaths were directly caused by diabetes mellitus. The International Diabetes Federation (IDF) estimated that almost 425 million people had DM in 2017, and this number is expected to increase to 629 million by 2045. DM claimed approximately 4 million adult deaths in 2017. Diabetes is also a major cause of blindness, kidney failure, heart attacks, stroke, lower limb amputation and other long-term consequences that significantly impact quality of life [2]. The prevalence of diabetes is gradually increasing in all age groups, particularly in middle-aged people, and is higher in low- and middle-income countries than in high-income countries. This increase is associated with the increasing incidence of overweight and obesity as well as population aging. An increase in deaths related to chronic hyperglycemia is being observed, along with an increased incidence of hyperglycemia [2, 3]. The disease reduces the mean life expectancy by 10–15 years on average. Angina pectoris, stroke, and atherosclerosis of the lower extremities are found earlier and more often in patients with diabetes, as a result of vascular complications; adults with diabetes have a two- to three-fold increased risk of heart attacks and strokes [4, 5]. Vascular complications in diabetes affect patients’ daily physical capacity and quality of life. Diabetes belongs to a group of disorders that reduce typical physical capacity and may influence pulmonary function [6].

The test of the ability to walk a defined distance is a quick, inexpensive and reproducible component of life quality and exercise capacity assessments. The six-minute walk test (6MWT) is currently widely used because it is both simple and reliable as a measure of exercise capacity. This test is a simple examination that assesses physical capacity and is readily accepted by patients [7]. The main advantage of simple exercise tests is that they do not require equipment and may thus be performed in most medical healthcare centers. The 6MWT plays a key role in evaluating functional exercise capacity, assessing prognosis and evaluating response to treatment. The 6MWT is most often used for the estimation of physical capacity in pulmonary and circulatory diseases in mild and severe conditions [8–10]. The test measures the six-minute walking distance (6MWD), while the walking pace depends on the patient. The test is performed once (single measurement) or before and after the treatment period or medical intervention (multiple measurements), as necessary. The 6MWT allows the estimation of the maximal exercise capacity of patients, which corresponds to the ability to perform daily activities. The 6MWT is an alternative to the maximal exercise test. The 6MWT can also be performed in elderly persons who have contraindications against tests on a cycle ergometer or treadmill [7, 11].

The second important point is that diabetes mellitus is associated with abnormal lung function and reduced gas transfer [12]. Chronic hyperglycemia directly increases nonenzymatic protein glycation, leading to the production of reactive oxygen species (O_2_^−^). Advanced glycation and oxidative stress trigger a chronic inflammatory response. Moreover, hyperglycemia is an important factor in the initiation and progression of microvascular complications in diabetes. Histopathological studies have shown an association between diabetes mellitus and the thickening of the alveolar epithelial basal lamina [13]. In this situation, diabetic patients can develop obstructive or restrictive patterns of respiratory disorders. On the other hand, as a result of alveolar-capillary membrane thickening due to diabetic microangiopathy and alterations in collagen and elastin, the capacity for the diffusion of carbon monoxide (DLCO) is reduced [14]. Despite strong evidence of a pathological effect of diabetes mellitus on the lung, it remains unclear whether this effect is clinically significant.

The research aimed to compare functional exercise capacity and pulmonary functions between patients with diabetes and healthy persons.

## Methods

### Group demographics

The diabetic patients were recruited among patients from diabetes clinics in the Tricity area (Gdansk, Gdynia, and Sopot), Poland. The eligibility criteria included the following: a diagnosis of type 1 or 2 diabetes mellitus, a duration of diabetes of at least one year, continued previous treatment for diabetes or its complications according to the international guidelines (oral hypoglycemic drugs and insulin medications). The control group consisted of healthy individuals from a general practice. The study included never-smokers over 18 years old. None of the participants had acute or chronic pulmonary or connective tissue disorders affecting pulmonary function. A medical history was taken from the patients as well as from the referring doctors (diabetes duration, glycemia control, medication used, concomitant diseases, diabetic complications, etc.). A physical examination was performed according to the protocol routinely applied in the Department of Allergology and Pneumonology. Special attention was paid to the measurements of body weight and height, body mass index (BMI, kg/m^2^), blood pressure and pulse rate while qualifying the patients for the study. Cardiovascular parameters, including heart rate and systolic and diastolic blood pressure, were measured in a sitting position using a digital sphygmomanometer (Omron Intelli Sense, Japan). Physical examinations of the chest wall and abdomen were also performed to assess health status and exclude possible acute disorders affecting the current state of the respiratory system.

All the participants obtained information in writing concerning the study and subsequently gave written consent to participate. The study was approved by the Independent Bioethics Committee of Scientific Research at the Medical University of Gdansk, Poland (NKEBN/14/2006).

### Experimental design

In both groups, blood tests (complete blood count, biochemical analysis, C-reactive protein (CRP), fibrinogen, and glycated hemoglobin (HbA_1c_)), spirometry, lung diffusing capacity for carbon monoxide (DLCO), whole body plethysmography and 6MWT were performed. The following parameters were measured: vital capacity (VC), forced expiratory volume in one second (FEV1), total lung capacity (TLC), DLCO and lung diffusing capacity for carbon monoxide/alveolar volume (DLCO/VA). A Lung Test 1000 spirometer with a lung diffusion module and body plethysmography chamber (MES *Poland*) was used for all measurements. All lung function tests were performed in a sitting position. The lung function tests were performed in accordance with the current American Thoracic Society/European Respiratory Society (ATS/ERS) guidelines [15, 16].

### Assessment of physical capacity

The 6MWT was used to assess functional capacity in this study. The 6MWT was carried out according to the current ATS guidelines [7]. The test was performed in a hospital corridor on a flat hard surface of 25 m in length in a straight line. Before the examination, the patient rested for 10 min in a sitting position. The technician supervising the test instructed the subject on the walking technique and the possibility of taking a rest. At one-minute intervals during the test, the participant received information about the time remaining until the end of the test. The blood pressure in the arm was measured before and after the 6MWT. During the whole test, blood saturation was constantly measured with a mobile pulse oximeter (OXY-Test 500, MES *Polan*d). The results of the blood pressure measurement, the peripheral blood oxygen saturation, increase in dyspnea and the distance walked in meters were noted on a case report form.

### Statistical analysis

The statistical data were divided into two groups: qualitative and quantitative. Patients with diabetes and healthy individuals, as well as subgroups of patients with diabetes (type 1 and 2), were compared. Moreover, the frequency of micro- and macroangiopathies was compared between the individual subgroups of diabetic patients.

In the statistical analysis, the χ^2^ test, t-test and analysis of variance (ANOVA) were applied. The χ^2^ test evaluates the independence and accuracy of fit of qualitative variables. A t-test was used for independent samples to test the significance of differences between mean values of the quantitative variables. The normal distribution of the data was confirmed using the Kolmogorov–Smirnov test. In the case of comparisons of more than two groups, continuous variables were analyzed with ANOVA. Statistical significance was defined as *P* < 0.05. Data analysis was carried out using Statistica Data Miner + QC software (StatSoft, USA).

## Results

### Group demographics

The study included a total of 131 participants: 64 patients with type 1 and 2 diabetes (DM) and 67 healthy patients in the control group (CG). The mean (SD) duration of diabetes was 13.2 (8.8) years. Forty-six diabetic patients routinely took insulin (all patients with type 1 diabetes and 22 patients with type 2 diabetes). The basic data concerning the examined groups are shown in Table 1.

**Table 1:** Demographic and clinical characteristics of the study groups

Variable	Control group (CG)	Diabetes mellitus group (DM)	*P* value
Number of persons	67	64	–
Females, *n* (%)	24 (35.8)	33 (51.6)	0.098
Males, *n* (%)	43 (64.2)	31 (48.4)	
Age (years)	18–75	18–83	0.001
Mean age (years)	41.2	51.6	
Height (cm)	155–192	153–194	0.05
Mean height (cm)	172.2	168.5	
Weight (kg)	54–130	53–140	0.062
Mean weight (kg)	81.4	86.4	
BMI (range)	19–43	18–45	0.01
Mean BMI	27.3	30.3	
CRP (SD)	1.6 (1.4)	4.8 (5.1)	0.001
Fibrinogen (g/l) (SD)	2.9 (0.7)	3.9 (0.9)	0.001
Microalbuminuria (mg/l) (SD)	–	63.6 (44.9)	–
Glycosuria (mg/l) (SD)	–	563.1 (299.9)	–
HbA_1c_ (%) (SD)	5.4 (0.3)	8.4 (2.0)	0.001
HbA_1c_ (mmol/mol) (SD)	36 (3.3)	68 (21.9)	0.001

The examined groups did not differ in terms of sex and weight. The people in the CG were taller than the patients with DM. The average body weight, average BMI and average age were higher in the DM group than in the CG. This finding is most likely related to higher concomitant obesity in people with diabetes, especially elderly people. Auscultation over the lung fields and heart showed no significant abnormalities.

The number of micro- and macroangiopathic complications in DM was thoroughly analyzed. Most patients, irrespective of the type of diabetes, suffered from chronic complications of diabetic angiopathy. No significant differences were found in concomitant microangiopathic complications in the examined groups of patients with diabetes. However, more complications related to macroangiopathy were observed in the group with type 2 diabetes. Hypertension was observed particularly often (85%) in this group of patients. Nephropathy, retinopathy and diabetic polyneuropathy were found with the same prevalence in both DM study groups (Table 2).

**Table 2:** Diabetes mellitus complications depending on types of diabetes mellitus

Complications of diabetes mellitus	Number of complications, *n* (%)	Type of diabetes mellitus		*P* value
		Type 1, *n* (%)	Type 2, *n* (%)	
Microangiopathy	40 (62.5%)	17 (70.8%)	23 (57.5%)	0.22
Macroangiopathy	46 (71.8%)	13 (54.7%)	33 (82.5%)	0.05
Microalbuminuria	22 (34.3%)	12 (50.0%)	10 (25.0%)	0.05
Nephropathy	19 (29.6%)	10 (41.6%)	9 (22.5%)	0.09
Polyneuropathy	17 (26.5%)	7 (29.1%)	10 (25.0%)	0.63
Retinopathy	25 (39.0%)	12 (50.0%)	13 (32.5%)	0.09
Coronary disorders	25 (39.0%)	6 (25.5%)	19 (47.5%)	0.08
Myocardial infarction	6 (9.3%)	1 (4.1%)	5 (12.5%)	0.37
Arterial hypertension	47 (73.4%)	13 (54.1%)	34 (85.0%)	0.01
Diabetic foot	4 (6.25%)	1 (4.1%)	3 (7.6%)	0.56

*n* number of complications

### Assessment of physical capacity and tests of pulmonary function

**Fig. 1:**
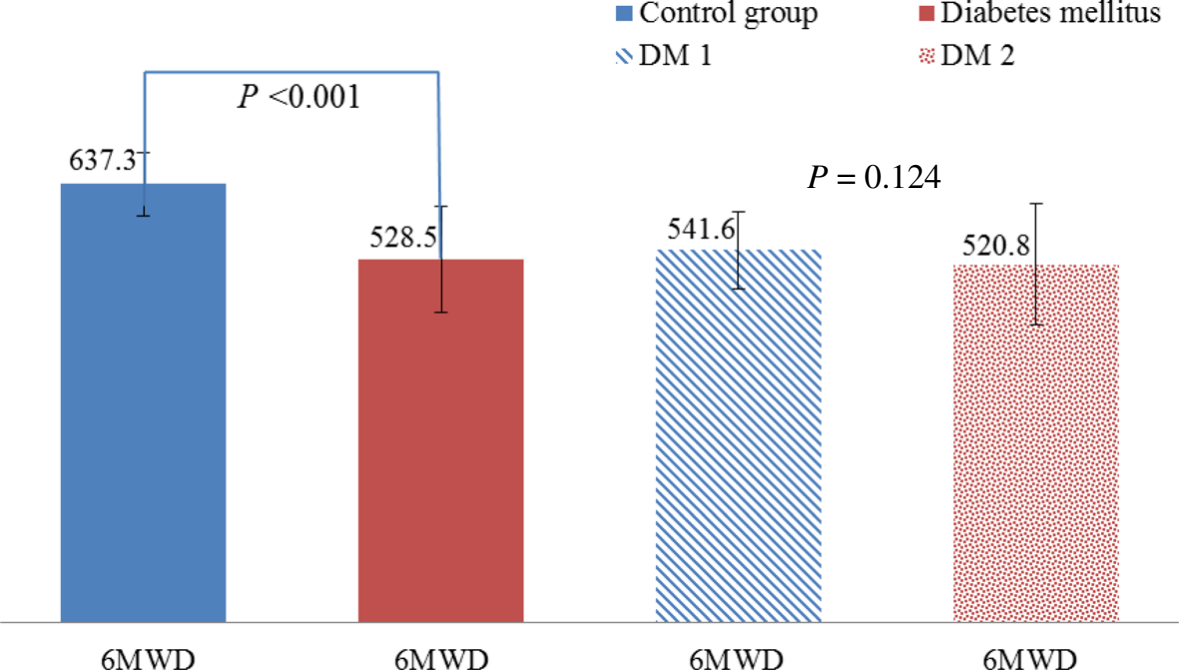
Results of 6MWT in diabetic and nondiabetic participants

The 6MWT, full spirometric tests, whole body plethysmography and DLCO were performed in both examined groups (Fig. 1, Table 3). The groups varied in terms of the distance covered. The average 6MWD was over 109 m shorter in the DM group than in the CG group (*P* < 0.001). The 6MWD did not differ significantly among the patients with different types of diabetes. The test was well tolerated by the participants. During the walking test, there were no significant adverse effects. None of the participants reported any pain of a coronary nature during or after the walking test. No dyspnea that prevented the test from continuing was observed in any of the groups. The most often reported symptoms included general fatigue, muscle pain and pain in the lower extremities as well as an uncomfortable feeling while walking. During and after the 6MWT, no significant reduction in oxygen saturation was found in the study groups.

**Table 3:** Baseline characteristics of diabetic and nondiabetic participants included in the study

Parameter	Control group *n* = 67	Diabetes mellitus *n* = 64	*P* value
VC (l)	4.9 (0.9)	3.9 (1.0)	0.001
VC (%)	114.4 (13.8)	105.2 (17.9)	0.001
FEV1 (l)	3.6 (0.7)	2.8 (0.8)	0.001
FEV1 (%)	103.0 (12.0)	94 (14.6)	0.001
TLC (l)	6.6 (1.1)	5.6 (1.2)	0.001
TLC (%)	106 (13.0)	99.5 (15.4)	0.001
DLCO (mmol/min/kPa)	10.0 (2.1)	8.6 (2.4)	0.001
DLCO/VA (mmol/min/kPa/l)	1.56 (0.2)	1.55 (0.2)	0.496

*n* number of measurement tests

The data are presented as the means (SDs)

In the study groups, some significant differences were found in the volume, capacity, pulmonary flow and diffusing capacity for carbon monoxide. The values of VC, TLC, FEV1, DLCO were significantly higher in the CG than in the DM group (Table 3).

## Discussion

### 6MWT and diabetes mellitus

This study was designed to investigate the functional exercise capacity in patients with DM and healthy controls. The findings from this study show that patients with DM demonstrated significantly lower exercise capacity than healthy controls. The 6MWT is widely used to measure the functional status and prognosis in patients with a wide variety of diseases, such as chronic obstructive pulmonary disease (COPD), pulmonary hypertension, and congestive heart failure. This test can also be utilized to investigate the effects of several interventions, for example, rehabilitation, treatment regimen changes, and oxygen supplementation, on patients’ walking capacity*.* In the literature, there are few papers that assess the use of the 6MWT in diabetic patients. Diabetes belongs to a group of disorders that reduce regular physical capacity [6, 17]. For example, Janevic et al. [18] found a significant reduction in the distance walked during the walking test in elderly women with diabetes (> 60 years old) with concomitant heart failure. Similar observations were made by other authors [19, 20] for patients with diabetes and congestive heart failure problems. In addition, Ingle et al. [21] observed a reduction in the distance walked in patients with diabetes and coexistent left ventricular systolic dysfunction (LVSD) during the walking test. Furthermore, these researchers noted that diabetes is an additional factor reducing the distance walked irrespective of coexisting LVSD. The poor exercise capacity in patients with diabetes based on the results of the 6MWT might be because of the link between diabetes and adverse cardiac effects. Thus, low exercise capacity may also have adverse consequences for DM patients during exercise [22]. Impaired exercise capacity is a powerful and independent predictor of an increased risk of cardiac events in DM patients. The reduction in exercise capacity in patients with DM may be linked to poor glucose metabolism. Hyperglycemia directly increases protein glycation and the formation of advanced glycation end products. In this situation, poor glycemic control has been associated with increased stiffness of vessels in the vascular bed in several organs, including the lungs [23]. Compliance plays a significant role in modulating coronary artery blood flow, which has important consequences for myocardial work capacity and, therefore, leads to reduced exercise capacity. A reduction in the distance walked in the walking test was also observed in patients with diabetes and concomitant peripheral circulatory insufficiency [24, 25]. Another important aspect of long-term diabetic complications is the involvement of the autonomic nervous system in almost every organ, including the lungs. Diabetic neuropathy can involve the entire autonomic nervous system—the vasomotor, visceromotor and sensory fibers innervating each organ. It has been shown that dysfunction of the cholinergic system [26] and adrenergic denervation [27] are significant parts of the clinical picture of diabetic neuropathy. In 2010, van Stolen et al. [28] published a paper assessing the influence of diabetic neuropathy on daily physical capacity. In the cohort of 100 people with type 2 diabetes, 40 people suffered from diabetic neuropathy, while obesity was present in 53 individuals (BMI > 30 kg/m2). The average 6MWD in the people with diabetes was 537 (SD 89) m. Daily activity was assessed by measuring the number of steps walked. The people with diabetes took 6500 steps a day, which was much fewer than recommended. It is believed that daily activity should amount to 10,000 steps per day [29]. Peripheral neuropathy and obesity were strictly related to the reduction in physical capacity in people with diabetes.

In this study, the groups varied in terms of the distance walked. The diabetic patients had poorer tolerance of physical exercise than the healthy controls. On average, the patients with diabetes covered a distance more than 109 m shorter than that covered by individuals in the CG. Importantly, a change of 30 m or more is considered a clinically important difference [11]. The difference in the 6MWT distance between the two groups can be partially explained by the fact that the patients with diabetes were significantly older, with a higher body weight and BMI, and showed micro- and macroangiopathic complications of diabetes. The number of diabetic complications was similar in both subgroups. The conditions for the 6MWT are similar to natural conditions in which a physical workload is present. In this study, the walking test was shown to be very useful in diversifying both groups (CG and DM) and determining daily physical capacity in the DM group. The usability of the 6MWT results from its direct significant connection with stress, heart rate, blood oxygen saturation and exertional dyspnea compared with the connection between these parameters and exercise tests on a cycle ergometer or treadmill in middle-aged adults [30, 31] and in elderly people [32]. It was also found that in elderly people of ages ≥68, the 6MWT correlates well with many characteristics and concomitant disorders [6]. The factors that significantly influenced the reduction in the 6MWT distance in both sexes included old age; higher body weight and abdominal circumference; lower muscle strength; the presence of depressive syndromes, joint diseases, coronary heart disease, stroke, or diabetes; lower education level; non-Caucasian race; angiotensin-converting enzyme inhibitor use; higher concentrations of fibrinogen; and higher white blood cell (WBC) count [33]. In women, a reduction in the 6MWT distance was also associated with low values of FEV1, diastolic blood pressure, and increased CRP concentration. Furthermore, the intake of digitalis resulted in a worsening of the results in men.

### Pulmonary function tests and diabetes mellitus

Pulmonary complications in diabetes attract the attention of researchers from various clinical disciplines, including an increasing number of pulmonologists. However, despite the regular updating of the relatively extensive literature with new scientific reports, pulmonary complications in diabetes remain difficult to explain. The lungs are a target organ for damage in diabetes, in which the transfer capacity of CO is decreased. The results of previous studies demonstrate that diabetes is independently associated with a clinically significant burden of respiratory symptoms and a reduction in lung function. The impact of diabetes on the lungs is confirmed by histopathologic examinations of the lung parenchyma and pulmonary function testing of the respiratory tract [34–36]. The studies show variations in the results obtained from spirometry, body plethysmography and the test of the diffusing capacity for carbon monoxide, which impedes a clear interpretation of the obtained results [37]. Moreover, most functional studies of the respiratory tract include nonsmokers without concomitant pulmonary disorders. On the other hand, concomitant obesity, tobacco smoking, heart failure and disorders related to diabetes can significantly influence the reduction in pulmonary function in patients with diabetes, especially those with type 2 diabetes mellitus [38]. However, no commonly acceptable and reproducible methods to identify and monitor pulmonary angiopathy have been introduced so far. One of the reasons for this reduction in pulmonary function may be the vascular and capacitive reserve of the lung parenchyma. This reserve compensates for the partial loss of ventilation and vascular reserves of the pulmonary circulation in the course of diabetes. Attempts to introduce inhalatory insulin for general use may provide further incentive for research on the course of pulmonary microangiopathy caused by chronic hyperglycemia [39, 40].

An interesting aspect of this study is the close concomitance of the lack of correction of metabolic parameters (HBA_1c_), the increase in biochemical parameters of chronic inflammation (CRP and fibrinogen) and the impairment of respiratory mechanics in diabetic patients. Compared with the CG, the patients with diabetes showed significant impairment of respiratory mechanics [VC (*P* < 0.05), FEV1 (*P* < 0.001), TLC (*P* < 0.05), DLCO (*P* < 0.001)].

It was also found that diabetes clearly influences the decrease in the strength and resistance of respiratory muscles, primarily the diaphragm [41]. The disorder also adversely affects the structure of collagen in the lung parenchyma and cartilage in the chest wall [42, 43]. These changes lead to the reduction in chest mobility [44] and thus to an additional deficiency in breathing mechanics. This progression suggests the systemic influence of diabetes, particularly on the respiratory system and indirectly on the physical capacity of these patients.

The present findings must be interpreted with caution because of some methodological limitations. One important limitation was the limited sample size. Another potential limitation of our study could be the presence of a number of existing underlying pathologies, such as cardiovascular disease, and a previous history of physical inactivity and an unhealthy lifestyle, which contribute to a low functional exercise capacity. Another caveat is that people with type 1 and type 2 diabetes were coanalyzed. However, both type 1 and type 2 diabetes have been associated with microangiopathy and macroangiopathy. The observations in this study included only diabetic patients who had never smoked and did not have coexisting chronic respiratory disorders. The influence of smoking and respiratory diseases can be additional factors adversely affecting the functional status of the respiratory system. Passive smoking has not been studied, despite its adverse effects on exercise capacity. One of the major limitations of 6MWT is the lack of adequate information on the mechanism and cause of disability during exercise. The distance walked in the 6MWT may vary according to the patient’s motivation or other individual factors. It should be noted that there are other factors affecting the result of the 6MWT: the patient’s motivation to exercise, leg length, and level of physical activity. Therefore, further studies with a larger sample size and a variable profile of diabetic subjects are recommended.

## Conclusion

Our study clearly demonstrates the difference in pulmonary function and the capacity for physical exertion between patients with DM and individuals in the CG. The mechanism of impaired exercise capacity is unclear but appears to be associated with the presence of diabetes. In conclusion, it should be noted that the 6MWT is a valuable test that complements the assessment of daily physical capacity in patients with diabetes, irrespective of type. The study demonstrated that diabetes is an independent factor reducing the distance walked, especially in patients with impaired respiratory mechanics. Pulmonary function and the capacity for physical exertion varied between the patients with diabetes mellitus and the healthy participants in the control group.

## Abbreviations

6MWD: six-minute walking distance
6MWT: six-minute walk test
CRP: C-reactive protein
DLCO: lung diffusing capacity for carbon monoxide
DLCO/VA: lung diffusing capacity for carbon monoxide/alveolar volume
DM: diabetes mellitus
FEV1: forced expiratory volume in one second
HbA1c: glycated hemoglobin
LVSD: left ventricular systolic dysfunction
TLC: total lung capacity
VC: vital capacity
WBC: white blood cell
